# The Effect of a Tele-Health Intervention Program on Home-Dwelling Persons with Dementia or MCI and on Their Primary Caregivers during the Stay-at-Home-Order Period in the COVID-19 Pandemic Outbreak: Evidence from Taiwan

**DOI:** 10.3390/healthcare10060969

**Published:** 2022-05-24

**Authors:** Yi-Ling Lai, Wen-Yi Chen, Lin-Ying Hsu, Chin-Hua Fu

**Affiliations:** 1 Community Health Center, Taichung Tzu Chi Hospital, Buddhist Tzu Chi Medical Foundation, Taichung 427213, Taiwan; ling@tzuchi.com.tw (Y.-L.L.); tc1370502@tzuchi.com.tw (L.-Y.H.); fu25588@ms58.hinet.net (C.-H.F.); 2 Department of Senior Citizen Service Management, National Taichung University of Science and Technology, Taichung 403301, Taiwan; 3 Department of Medicine, Buddhist Tzu Chi University, Hualien 970374, Taiwan

**Keywords:** Tele-Health, dementia, mild cognitive impairment, stay-at-home-order policy, lockdown, COVID-19, SARS-CoV-2

## Abstract

Background: The Taiwanese government implemented a stay-at-home order that restricted all community-based health promotion activities for the elderly by shutting down all community care centers from May 2021 to August 2021 to control the spread of COVID-19. Community-based dementia care centers were barely able to provide dementia care services during that period. Methods: The data used in this study were collected from a community-based dementia care center that was able to continue their dementia care services through a Tele-Health intervention program. The difference-in-differences methodology was applied to evaluate the effects of the Tele-Health intervention program on home-dwelling persons with dementia or mild cognitive impairment and on their primary caregivers during the COVID-19 pandemic. Results: The Tele-Health intervention program significantly increased the well-being of the participants and their primary caregivers, but the negative correlations between the Tele-Health intervention program and family functioning were also found to be significant. Conclusions: The significant substitution (negative) effects between the Tele-Health intervention program and family functioning raises the concern that promotion of the Tele-Health intervention program comes at the potential cost of a loss of family functioning. Policymakers should be cautious when considering the Tele-Health intervention program in response to pandemics and demographic transitions.

## 1. Introduction

SARS-CoV-2 (COVID-19) has forced most countries worldwide to implement many public health interventions (e.g., face mask wearing, social distancing practices, school closures, quarantines, and lockdowns) to constrain the spread of COVID-19 [1,2]. Although these interventions have effectively controlled spread, the subsequent adverse effects on persons with dementia have been well documented in the literature. For example, Suárez-González and her colleagues provided a rapid systematic review to examine the effects of the COVID-19 isolation measures on the cognition and mental health of persons with dementia [3]. The findings generated from the 15 selected eligible studies suggested that lockdowns are associated with a worsening of cognition, of behavioral and psychological symptoms, and of the level of activities of daily living functions in persons with dementia. Additionally, Soysal and her colleagues conducted rigorous meta-analyses to investigate the effects of lockdowns on neuropsychiatric symptoms in persons with dementia or mild cognitive impairment (MCI) [4]. Their review identified 21 eligible studies published since June 2021, and the results obtained from their meta-analyses indicated an increase in the worsening of neuropsychiatric symptoms in persons with dementia or MCI during the lockdowns of the COVID-19 pandemic [4]. The same results can be found under other strategic interventions to control COVID-19 community spread, such as social distancing practices [5], quarantines [6,7], and social support service closures [8,9].

Since caregivers of persons with dementia or MCI are at risk of psychological burden and physical strain due to the loss of a social support network (or supportive care services), isolation increments, and the instability of neuropsychiatric or behavioral and psychological symptoms in their care recipients during the COVID-19 pandemic [10,11], there is another strand of the literature exploring the association between the COVID-19 outbreak and the wellbeing level of caregivers of persons with dementia around the world. A significantly negative impact of the COVID-19 pandemic on the wellbeing of caregivers of persons with dementia or MCI has been found in Argentina [12,13], Brazil [12,14], Chile [12], China [15], France [16], Greece [17,18], Italy [19,20,21], Spain [22], Switzerland [19], and a group of OECD countries [23].

In response to the adverse effects of the COVID-19 pandemic on both persons with dementia and their primary caregivers as indicated by the aforementioned literature, many researchers have provided support for the Tele-Health intervention program, which digitally delivers supportive care services to persons with dementia or MCI and their primary caregivers, as a policy instrument designed to mitigate such effects [24,25,26]. Nevertheless, previous studies exploring whether or not the Tele-Health intervention program could benefit persons with dementia, or MCI and their caregivers, have demonstrated ambiguous results. Some studies found little or no effect of the Tele-health intervention program on caregivers’ burdens, depressive symptoms, and health-related quality of life [27], and found insignificant improvements in objective caring skills [28]. Conversely, other research revealed that the Tele-Health intervention program not only improves quality of life and ameliorates neuropsychiatric symptoms in persons with dementia or MCI, but also leads to a better health outcome for primary caregivers of persons with dementia or MCI, a lower risk of mental health impairment [29,30,31,32], burden reduction [29,33], and improvements in knowledge of dementia [29].

Given the need for continuous care delivery to those requiring dementia care services during the COVID-19 pandemic, evaluations of the effects of the Tele-Health intervention program on persons with dementia or MCI and their primary caregivers has become an important subject for policymakers that seek to design new healthcare delivery models under the potential threat of future pandemics. In this study, we specifically investigate the effects of the Tele-Health intervention program on home-dwelling persons with dementia or MCI and their primary caregivers (referring to an unpaid family member carrying out daily tasks such as handling household chores and preparing meals and medication) during the Stay-at-Home-Order period of the COVID-19 pandemic (i.e., the period from May 2021 to August 2021) in Taiwan. The significance of this study is threefold: first, Taiwan has one of the fastest-aging populations in the world. The proportion of the Taiwanese elderly (aged 65 and higher) is higher than 14%, a threshold in accordance with the UN definition of an aged society since 2018, and Taiwanese society is anticipated to become a hyper-aged society by 2027, according to the projection that the elderly population (aged 65 and higher) will occupy more than 21% of the total population in 2027, a threshold compliant with the UN definition of a hyper-aged society [34]. Thus, the demographic transition from an aged society to a hyper-aged society will only take 10 years in Taiwan, and there is a high risk that this demographic transition will dramatically impact its society. Second, approximately 26% of the Taiwanese elderly population was diagnosed as having mild or worse impairment in their intellectual function in 2021 [35]. In addition, the Taiwanese elderly population with dementia is approximately 296,400 (equivalent to a 7.71% dementia prevalence rate among this demographic) in 2021, and this number is expected to rise to 660,100 (equivalent to a 9.51% dementia prevalence rate) in 2041 [35]. This 2.28-fold increase in the total elderly population with dementia within a 20-year span is much higher than the expected rise in the world average (about 2.0) [36], so the burden of dementia imposed on Taiwan’s healthcare system is expected to be substantial. Third, the Tele-Health intervention program has advantages in terms of the prevention of COVID-19 infection and delivers timely supportive care services to home-dwelling persons with dementia or MCI and their primary caregivers. It follows that it is essential for researchers and policymakers to determine the beneficial effects of the Tele-Health intervention program in light of the potential threat of future pandemics.

It is important to acknowledge that there are two major challenges in evaluating the effects of the Tele-Health intervention program on home-dwelling persons with dementia or MCI and their primary caregivers in Taiwan. First, according to the Taiwan’s long-term care policy based on the active ageing and ageing in place guidelines [37], supportive care services for home-dwelling persons with dementia or MCI and their primary caregivers are encouraged to be provided on-site in community-based dementia care centers. In general, there was no Tele-Health intervention program (i.e., supportive care services digitally delivered to persons with dementia or MCI and their primary caregivers) implemented for home-dwelling persons with dementia or MCI and their primary caregivers before the COVID-19 pandemic. Second, the Taiwanese government offers inexpensive community-based care services for elderly residents, so home-dwelling persons with dementia or MCI and their primary caregivers usually participate in many on-site health intervention programs simultaneously. Multiple participation in health intervention programs among the Taiwanese elderly population makes it difficult to justify a single intervention program for home-dwelling persons with dementia or MCI and their primary caregivers owing to the mixed effects involved with multiple-intervention programs.

In response to the challenges of evaluating the effects of the Tele-Health intervention program in Taiwan, we studied the Stay-at-Home-Order policy implemented by the Taiwanese government from May 2021 to August 2021. This policy was implemented to protect the elderly population from COVID-19 infection by shutting down all community-based care centers, through which various community care services for the elderly were delivered. In addition, this policy also restricted visits to institutionalized inhabitants in long-term care facilities and hospitals. It followed that community-based dementia care centers were barely able to provide any services during the Stay-at-Home-Order period (i.e., May 2021 to August 2021) in Taiwan. In order to demonstrate the beneficial effect of the Tele-Health intervention program, we collected data from a community-based dementia care center capable of continuing their dementia care services through the Tele-Health intervention program (i.e., digitally delivering supportive care services to persons with dementia and their caregivers). Note that the Stay-at-Home-Order policy eliminated a bias from the mixed effects of elderly residents’ participation in many health intervention programs. The difference-in-differences (DID) methodology introduced by Chen and her colleagues [38] was applied to evaluate the effects of the Tele-Health intervention program on persons with dementia or MCI and their primary caregivers during the COVID-19 pandemic. The results generated from this study could serve as an important reference for policymakers to evaluate whether the Tele-Health intervention program is an appropriate policy instrument for delivering various supportive care services to persons with dementia or MCI and their primary caregivers.

## 2. Materials and Methods

### 2.1. Context of the Study

Taiwan’s long-term care system is financed by various types of taxes (such as tobacco, estate, and gift taxes) and the central government’s budget, and it is a universal coverage long-term care plan providing four categories of long-term care services, including (1) personal and professional care (such as home and daycare services, home nursing, rehabilitation, nutrition, and among others), (2) transportation, (3) assistive devices and home modifications, and (4) respite care for all residents in Taiwan. The long-term care delivery system was built on a community-based integration network of three-layered care providers (termed as the ABC network). The Type A providers play the role of the individual’s long-term care planner who integrates care services provided by the Type B and Type C providers for their care recipients. The missions of the Type B and Type C providers are to provide personal and professional long-term care services and to stimulate community-health promotion activities for maintaining the elderly’s physical and intellectual functions, respectively. A community-based dementia care center administrated by a hospital could be classified as one of the Type B providers in Taiwan.

It is important to address that community-based dementia care centers were scarcely able to provide dementia care services through the Tele-Health intervention program in Taiwan during the Stay-at-Home-Order period (i.e., the period of May 2021–August 2021) due to a lack of resources including necessary facilities, well-trained staff, and financial support. Nevertheless, we collected our research data from a community-based dementia care center where an experimental Tele-Health intervention program had been developed in response to the potential threat of future pandemics. The dementia care center under examination is administrated by Taichung Tzu Chi Hospital, a regional teaching hospital providing tertiary care and supporting teaching and research in clinical practices in the middle of Taiwan. Prior to the Stay-at-Home-Order period, this dementia care center functioned as a daycare center accommodating 33 elderly individuals diagnosed as having MCI or dementia, and it provided long-term care services, including health promotion activities, for the maintenance of physical and intellectual functions and programs designed to increase primary caregivers’ competence and ability to cope with challenging situations and to relieve their feelings of isolation and their burdens of caring for persons with dementia or MCI. Note that the primary caregiver in this study refers to an unpaid family member carrying out daily tasks such as handling household chores and preparing meals and medication. All long-term care services provided by the dementia care center under examination were funded by the Taiwan Ministry of Welfare and Health (MOWH) before the COVID-19 pandemic.

### 2.2. Participants

Thirty-three elderly individuals receiving long-term care services were recruited from the dementia care center under examination. Those who had severe hearing and language impairment were excluded. Eighteen participants were recruited, and ten and eight participants were placed into experimental and control groups, respectively, based on their willingness to participate. One of these eighteen participants were diagnosed as having MCI, and other seventeen participants were diagnosed as having dementia. The participant with MCI and her primary caregiver (i.e., an unpaid family member carrying out daily tasks) chose to participate in the experimental group. Pre- and post-test interviews for primary caregivers together with participants were conducted by one staff member who is a registered nurse (RN) and has a master’s degree in public health (MPH). Two participants (diagnosed as having dementia) in the experimental group were further excluded from our data analyses because they did not participate in the pre-test interviews; therefore, 32 observations extracted from the pre- and post-test interviews of these 16 participants were used in our data analyses.

### 2.3. Procedures

The Tele-Health intervention program was operated by the following procedures: First, a one-hour synchronous distance teaching class for various health promotion activities was digitally delivered to participants in the experimental group twice a week, and telephone contact with participants or their primary caregivers in the control group also occurred twice a week. This Tele-Health intervention was an eight-week program, starting from May 2021 to August 2021 (i.e., the Stay-at-Home-Order period). Second, class materials were mailed to participants’ households one week before the synchronous distance teaching class began, and confirmation that class materials were received by participants was established by telephone. Moreover, the staff contacted the primary caregivers of participants approximately 10–30 min before the synchronous distance teaching class started in order to ensure the online connection of computers, communications, and consumer electronics from participants’ households to the webcam system installed on the main computer at the dementia care center. Third, all participants and their primary caregivers were invited to undergo two online surveys (pre-test and post-test interviews) with a structural questionnaire designed to investigate participants’ neuropsychiatric symptoms and their primary caregivers’ stress levels, their attitudes regarding technology acceptance, and their family functioning during the period of May 2021–August 2021. The Tele-Health intervention program was free of charge to all participants. Informed consent forms were signed by all participants and their primary caregivers. The data collection process was approved by the Research Ethics Committee of Taichung Tzu Chi Hospital with ID: REC109-22.

### 2.4. Instruments and Variables

We collected the demographic information (e.g., gender and age) of the participants and their primary caregivers. Since the Tele-Health intervention program relies largely on the participants’ primary caregivers, we gathered more individual information about these primary caregivers such as their marital status, their level of education, their attitude regarding technology acceptance, and their family functioning. Furthermore, participants’ neuropsychiatric symptoms and stress levels were recorded to evaluate the beneficial effect of the Tele-Health intervention program on participants and their primary caregivers. The operational definitions of variables used in this study are given below:

#### 2.4.1. Intensity of Neuropsychiatric Symptoms

Neuropsychiatric symptoms were measured by the neuropsychiatric inventory (NPI) questionnaire [39]. During the course of administering the NPI questionnaire, we first asked the primary caregiver to assess whether the participant had the following 10 behavioral symptoms: delusions, hallucinations, agitation/aggression, depression/dysphoria, anxiety, elation/euphoria, apathy/indifference, disinhibition, irritability/lability, and aberrant motor behaviors. If the answer was negative, the subscale of the intensity of that specific behavioral symptom was zero. Conversely, if the answer was positive, we then asked the primary caregiver to assess the severity and frequency of that specific behavioral symptom. Severity was rated on a three-point scale, ranging from one to three points corresponding to mild (i.e., the symptom produces little distress in the participant), moderate (i.e., the symptom is more disturbing to the participant but can be redirected by the primary caregiver), and severe (i.e., the symptom is very disturbing to the participant and difficult to redirect). In addition, frequency was rated on a four-point scale, ranging from one to four points corresponding to rarely (less than once per week), sometimes (about once per week), often (several times per week but less than every day), and very often (once or more per day). The subscale of the intensity of the single behavioral symptom was the product of severity and frequency. The intensity of neuropsychiatric symptoms was measured by the sum of all subscales of the intensity of these 10 behavioral symptoms.

#### 2.4.2. Stress Assessment

The Taiwan Association of Family Caregivers has developed a family caregiver stress survey (available online at https://www.familycare.org.tw/policy/10643, accessed on 18 April 2022) for the purpose of assessing a family caregiver’s stress level. This survey is frequently used for long-term care practitioners to measure a primary caregiver’s stress in a household, and it consists of 14 statements: Q1 (I feel that I still have to take care of him/her when I am not feeling well), Q2 (I feel exhausted), Q3 (I feel physically burdened), Q4 (I feel that I am affected by his/her emotions), Q5 (I feel that my sleep is disturbed because he/she cannot sleep well at night), Q6 (I feel that my health has suffered because of my involvement with him/her), Q7 (I feel burnout), Q8 (I feel that my involvement with him/her makes me mentally distressed), Q9 (I feel angry when I stay with him/her), Q10 (I feel that my travel plan is changed because of my involvement with him/her), Q11 (I feel that my social life with family and friends has suffered because of my involvement with him/her), Q12 (I feel that I have to keep an eye on him/her at all times), Q13 (I feel that the expense of taking care of him/her is high and burdensome), and Q14 (I feel that our household income is affected due to my taking care of him/her). The primary caregiver was asked to rate frequency based on a four-point scale, ranging from zero to three points corresponding to never, rare (about once more than a week), sometimes (about once per week), often (several times per week but less than every day), and very often (once or more per day). The sum of the subscale of these 14 statements was used to measure the primary caregiver’s stress level.

#### 2.4.3. Technology Acceptance Attitude

The primary caregivers’ attitude regarding technology acceptance was investigated through a revised version of the technology acceptance survey introduced by Davis and his colleagues [40,41]. Eleven statements with a five-point Likert scale ranging from “strongly disagree” to “strongly agree” were suggested by Chou and Lu [42] to measure four dimensions of their attitude regarding technology acceptance: the perceived usefulness of using a new healthcare delivery model (Q1: the synchronous distance teaching class enables me to improve my care skills; Q2: the synchronous distance teaching class enhances my effectiveness in caring for my family members; Q3: it is convenient for me to gain knowledge and care skills regarding dementia from the synchronous distance teaching class; Q4: overall, I find that the synchronous distance teaching class is useful in caring for my own family), the perceived ease of using it (Q5: I find it is easy to do what I want to do in the synchronous distance teaching class; Q6: interactions in the synchronous distance teaching class are easy for me to understand), their attitude toward using it (Q7: I think it is smart to participate in the synchronous distance teaching class; Q8: I think it is favorable for me to participate in the synchronous distance teaching class; Q9: I think participating in the synchronous distance teaching class is pleasant), and their intention to use it again (Q10: I will participate in the synchronous distance teaching class more frequently in the future; Q11: I am happy to participate in the synchronous distance teaching class in the future). Based on the Technology Acceptance Model (TAM) derived by Davis and his colleagues [39,40], the perceived usefulness and perceived ease of using technology are antecedent variables of the attitude toward technology, and the intention to use technology is likely to depend on the perceived usefulness, perceived ease of using, and attitude toward technology.

#### 2.4.4. Family Functioning Assessment

The caregivers’ family functioning was assessed using the Taiwanese family function questionnaire developed by Shiau and her colleagues [43]. Thirty-four questions were designed to measure ten dimensions of family functioning: problem solving, decision-making power, communication, affective, role, marital, healthcare, behavior control, dependence, and socialization. All questions were rated on a four-point Likert scale ranging from “rare” (occurrence rate below 25%), “sometimes” (occurrence rate between 25–49%), “often” (occurrence rate between 50–75%), and “very often” (occurrence rate higher than 75%). We extracted 16 questions measuring five dimensions of family functioning: problem solving (i.e., the capability of a family to solve problems and retain family functioning), communication (i.e., the information exchanged between family members), role (i.e., tasks assigned to family members to complete family functions), affective (family members showing concern and emotionally responding to each other), and behavior control (the maximum freedom of members in the family). The construct of these family functioning dimensions is akin to the Family Assessment Device based on the theoretical concept of the McMaster model of family functioning theory [44].

#### 2.4.5. Demographic and Intervening Variables

Age was computed by survey year (i.e., 2021) minus the year of birth. Several dichotomous variables were used to define demographic characteristics of the participants and their primary caregivers, such as gender (male = 1; female = 0), marital status (single = 1; others = 0), and education attainment (bachelor or higher = 1; others = 0). The intervening variables were also defined by dichotomous variables, including a *Case* dummy (experimental group = 1; control group = 0) and a *Time* dummy (post-intervention = 1; pre-intervention = 0), and an interaction dummy (i.e., *Case* × *Time*).

### 2.5. Statistical Analyses

The statistical analyses were conducted as follows: First, frequency distribution was demonstrated to describe the demographic characteristics and the distribution of each variable. Second, a chi-square test and a *t*-test were applied to determine the differences in proportions and the mean values of the variables or instruments between experimental and control groups, respectively. Third, the descriptive statistics for the DID of the target variables were reported. Fourth, due to a small sample size, control variables, such as demographic variables, and other instruments were selected by a stepwise regression procedure with a *p*-value less than 5%, and we then estimated the DID regression model with a random effect to incorporate potentially unobserved factors of participants and their primary caregivers (such as health status and household conditions). Robust standard errors based on the work of Chen and her colleagues [38] were used to compute T statistics in order to prevent biased inferences generated from the Ordinary Least Square (OLS) estimation.

More specifically, the DID regression model can be specified as follows:

(1) $$Y_{it}=\alpha +\beta_{E}Case_{i}+\beta_{T}Time_{t}+\beta_{C}{\left(Case\times Time\right)}_{it}+\gamma CV_{it}+\varepsilon_{it}$$

where, $Y_{it}$ represents the individual *i*’s target variables such as NPI intensity and stress level, technology acceptance attitude, and family functions at time *t*. $Case_{i}$, $Time_{t}$, ${\left(Time\times Case\right)}_{it}$, and $CV_{it}$ denotes the case dummy, time dummy, interaction dummy, and control variables, respectively. $\beta_{E}$, $\beta_{T}$, $\beta_{C}$, and γ are parameters corresponding to $Case_{i}$, $Time_{t}$, ${\left(Time\times Case\right)}_{it}$, and $CV_{it}$, respectively. α and $\varepsilon_{it}$ denote the constant and residual terms, respectively. Note that $\beta_{E}$ + $\beta_{C}$ ($\beta_{E}$) is the difference of target variables between experimental and control groups in the post-intervention (pre-intervention) period, and $\beta_{T}$ + $\beta_{C}$ ($\beta_{T}$) is the difference of target variables across the post- and pre-intervention period in the experimental (control) group; therefore, $\beta_{C}$ is the difference-in-differences of target variables, which is the target effect under study. The formal parametrization of the DID regression model is given by Table 1 below:

Since the analysis of covariance (ANCOVA) model is a popular method that is frequently used to evaluate the post-intervention outcomes between different groups (or treatments), we also demonstrate the specification of the ANCOVA model below:

(2) $$Y_{i}^{t}=\alpha^{\prime }+\beta_{E}^{\prime }Case_{i}+\beta^{\prime }Y_{i}^{t-1}+\gamma^{\prime }CV_{i}^{t-1}+\xi_{i}$$

where, $Y_{i}^{t}$($Y_{i}^{t-1}$) represents the individual *i*’s target variables at time *t*(*t* − 1). $Case_{i}$$CV_{i}^{t-1}$, and $\xi_{i}$ are the case dummy, control variables, and residual term, respectively. $\alpha^{\prime }$ is a constant term, and $\beta_{E}^{\prime }$, $\beta^{\prime }$, $\gamma^{\prime }$ are parameters corresponding to $Case_{i}$, $Y_{i}^{t-1}$, and $CV_{i}^{t-1}$, respectively. A stepwise regression procedure with a *p*-value less than 5% was applied to choose control variables, due to a small sample size.

It is important to note that the DID regression model is likely to be more suitable for this study than the ANCOVA model, for the following reasons: first, the results identified from the ANCOVA model concern whether the target variables in the post-intervention period were significantly different between experimental and control groups, whereas the purpose of this study is to gauge whether the changes of target variables across the Stay-at-Home-Order period were significantly different between experimental and control groups; therefore, it is essential to note that the insignificant results obtained from the ANCOVA models do not necessarily mean an ineffectiveness of the Tele-Health intervention program on home-dwelling persons with dementia or MCI and their primary caregivers. The target effect of a Tele-Health intervention program on home-dwelling persons with dementia and their primary caregivers should be identified by the parameter $\beta_{C}$ in the DID regression model rather than the parameter $\beta_{E}^{\prime }$ in the ANCOVA model.

Second, the panel data pooling pre- and post-intervention periods (32 observations in total) were used to estimate the DID regression model, whereas only 16 observations were used to estimate the ANCOVA model, due to the nature of cross-sectional model specification of the ANCOVA model; therefore, observations used in the ANCOVA model were 50% lower than those used in the DID regression model. It follows that the ANCOVA model was likely to encounter a more severe issue concerning a small sample size than the DID regression model. Third, since the ANCOVA model is not a repeated measurement model, it is unable to incorporate potentially unobserved factors of participants and their primary caregivers through a random effect imposed in the residual term.

## 3. Results

### 3.1. Descriptive Statistics

Table 2 presents the descriptive statistics of all variables relating to individual characteristics and instruments used in this study. As indicated in Table 1, all participants were equally split between males and females. Approximately 75% (=12/16) and 37.50% (=6/16) of primary caregivers were female and single, respectively. Approximately 75% (=12/16) of primary caregivers’ education attainment was beyond a bachelor’s degree. The mean values of the participants and their caregivers’ age are approximately 80 and 57, respectively. The chi-square test and the *t*-test showed that the differences in the characteristics of the participants and their primary caregivers between the experimental and control groups were not significant (i.e., *p*-values were all higher than 10%). These results indicate that the demographic characteristics of the participants and their primary caregivers were homogeneous across the experimental and control groups.

The mean values of the NPI intensity score and the stress score of the participants and their primary caregivers were 14.250 and 16.531, respectively. The *t*-tests for the differences in the NPI intensity and stress scores were found to be positively significant at the 10% level and insignificant, respectively. These results suggest that the participants in the experimental group had a significantly higher NPI intensity score than those in the control group, but the primary caregivers’ stress level was not different across the experimental and control groups. The mean values of the four dimensions of participants’ attitudes towards using a new healthcare delivery model, i.e., its perceived usefulness, the perceived ease of using, their attitude toward using it, and their intention to use it, were 16.000, 8.250, 12.563, and 8.438, respectively. The *t*-tests showed that the differences in the mean scores of these four dimensions between the experimental and control groups were significantly positive at the 10% (or rigorous) level, and this suggests that primary caregivers in the experimental group were more accepting of the technology than those in the control group. In addition, the mean scores of the five family functioning dimensions, corresponding to problem solving, communication, affective, role, and behavior control, were 8.313, 5.531, 15.875, 8.406, and 7.000, respectively. The *t*-tests showed that the differences in mean scores of these family functioning dimensions between the experimental and control groups were not significant, indicating that the family functioning of primary caregivers in the experimental group was similar to that in the control group.

### 3.2. DID in the Mean Values of the Target Variables

Table 3 presents the descriptive statistics for the DID of the target variables. As demonstrated in Table 3, the average stress score for primary caregivers in either the experimental or the control group showed a downward trend (see the negative values in the differences in mean values) over the Stay-at-Home-Order period. Nevertheless, the mean scores of the NPI intensity and the five family functioning dimensions decreased (i.e., negative values in the differences in mean values) in the experimental group, but we found either an upward or an invariable trend in the control group (i.e., positive values or zero in the differences in mean values) during the same period. Conversely, the mean values of the scores of the primary caregivers’ attitudes toward using a new healthcare delivery model and their intention to use it increased (i.e., positive values in the differences in mean values) in the experimental group, but we found a downward trend in the control group (i.e., negative values in the differences in mean values).

The DID in the mean scores of the stress scale, the NPI intensity, and the five family functioning dimensions were also negative, but those in the average scores of the primary caregivers’ attitudes toward using a new healthcare delivery model and their intention to use it were positive. These preliminary results illustrate that not only did the Tele-Health intervention program reduce participants’ NPI intensity and their primary caregivers’ stress, but it also made the primary caregivers more accepting of the technology during the Stay-at-Home-Order period. Nevertheless, several restrictions on the statistical inferences from Table 2 need to be addressed: first, the testing statistics of the DID in the mean values of the target variables cannot be generated directly from Table 3. Second, it is necessary to control the potential heterogeneity in the characteristics of the participants and their primary caregivers between the experimental and control groups, and some corrections in the standard errors of the DID in the mean values of the target variables should be made in case the assumptions of the residuals from the OLS estimation are violated [38].

### 3.3. Results for the ANCOVA Model

Table 4 presents our estimation results for the ANCOVA model. As shown in Table 4, F statistics for testing the null hypothesis of the indifference of target variables (such as NPI, stress level, technology acceptance attitude, and family functions) in the post-intervention period between experimental and control groups generated *p* values higher than 10%. These results suggest that there were no significant differences of target variables in the post-intervention period. Thus, we evaluated the target effect of the Tele-Health intervention program on home-dwelling persons with dementia or MCI and their primary caregivers through testing for whether the changes of target variables across the Stay-at-Home-Order period were significantly different between experimental and control groups. The estimated coefficient of an interaction dummy (i.e., *Case* × *Time*) from the DID regression model was used to gauge the target effect of the Tele-Health intervention program.

### 3.4. Results for the DID Regression Model

In order to conquer the restrictions on the statistical inferences from Table 3 and to justify the target effect of the Tele-Health intervention program, we estimated the DID regression models with a random effect to incorporate potentially unobserved factors affecting certain variables regarding the participants and their caregivers (such as health status and household conditions). As indicated in Table 5, the null hypotheses of normality in the residuals of all estimated DID regression models were confirmed because the *p*-values generated from the *JB* statistics for these models are higher than a 10% significance level. In addition, the *DW* statistics for all estimated DID regression models fall into ranges between the upper boundary of the *DW* statistics and four minus the upper boundary of the *DW* statistics at a 5% significance level, so the null hypothesis of non-serial correlation in the residuals from our estimated DID regression models cannot be rejected [45]. Nevertheless, the null hypotheses of homoscedasticity in the residuals were rejected in some of our estimated DID regression models since the *p*-values generated from the *LR* statistics were at a significance level of less than 10%. Thus, the robust standard errors based on the work of Chen and her colleagues [38] were used to compute T statistics.

As shown in Table 5, we found that the estimated coefficients of the interaction dummy (i.e., *Case* × *Time*) were significantly negative regarding the primary caregiver’s stress level and the NPI intensity of their care recipients. The DID mean scores of the stress and the NPI intensity generated from the DID regression models were −3.875 and −8.796, respectively, meaning that the participation in the Tele-Health intervention program decreased the primary caregivers’ stress score and the NPI intensity score of their care recipients by 3.875 (equivalent to 23.44% = 3.875/16.531 reduction in mean score) and 8.796 (equivalent to 61.73% = 8.796/14.250 reduction in mean score), respectively. The same significant results were found in the DID regression models of the five dimensions of family functioning. The estimated coefficients of the interaction dummy (i.e., *Case* × *Time*) were −2.250, −1.625, −3.211, −1.375, and −1.476, corresponding to the DID mean scores of the family functioning dimensions of problem solving, communication, affective, role, and behavior control, respectively. These results suggest that the participation in the Tele-Health intervention program reduced the scores of problem solving, communication, affect, role, and behavior control in family functioning by 2.250 (equivalent to 27.07% = 2.250/8.313 reduction in mean score), 1.625 (equivalent to 30.48% = 1.625/5.331 reduction in mean score), 3.211 (equivalent to 20.23% = 3.211/15.875 reduction in mean score), 1.375 (equivalent to 16.36% = 1.375/8.406 reduction in mean score), and 1.476 (equivalent to 21.09% = 1.476/7.000 reduction in mean score), respectively. Nevertheless, the estimated coefficient of the interaction dummy (i.e., *Case* × *Time*) was found to be significantly positive for the primary caregivers’ intention to use a new healthcare delivery model. The DID mean score of this intention was 0.721, meaning that the participation in the Tele-Health intervention program increased the score of this intention by 0.721 (equivalent to 8.54% = 0.721/8.438 increment in mean score). Participation in the Tele-Health intervention program did not generate any significant results in terms of the primary caregiver’s attitude toward using a new healthcare delivery model.

The control variables selected by our stepwise regression procedure included the primary caregiver’s marital status, gender, stress scale, their perceived usefulness, their perceived ease, their attitude toward using a new healthcare delivery model, the affect and behavior control in family functioning, and the participants’ age and NPI intensity. The primary caregivers whose marital status was single had lower levels of problem solving, communication, and behavior control in family functioning than their counterparts, but the primary caregivers who are male had a higher functioning role in their households than their counterparts. The participants’ age was negatively associated with the score of role function. The primary caregivers’ stress level was positively (negatively) associated with their intention to use a new healthcare delivery model (affective family function). The participants’ NPI intensity was negatively correlated with the behavior control family function. Moreover, the primary caregiver’s perceived usefulness of the new delivery model, their perceived ease of using it, and their affective family function were positively related to their attitude toward using the new delivery model.

## 4. Discussion

### 4.1. Policy Implications

Several policy implications obtained from our DID analyses need to be addressed. First, the beneficial effects of the Tele-Health intervention program on home-dwelling persons with dementia or MCI and their primary caregivers were confirmed due to a significant reduction in the participant’s NPI intensity and their primary caregiver’s stress level. These findings are consistent with results gained from previous studies on the effect of Tele-Health-based intervention on persons with dementia and caregivers [29,30,31,32,33]. Since the Tele-Health intervention program (digitally delivering supportive care services to households) protects against the infection of COVID-19, the development of more sophisticated Tele-Health intervention programs could be essential—given the potential risk of future pandemics—for the Taiwanese government to assure continuous care provisions for those who are in need of dementia care.

Second, it is important to address that only tangible (or on-site) dementia care services could be reimbursed from the government under Taiwan’s long-term care system. This condition created financial disincentives for long-term care providers to provide the Tele-Health intervention on the occurrence of significant outbreaks of infectious diseases. The priority setting for the future reform of Taiwan’s long-term care system could accentuate on the establishment of feasible standards to measure the quality of Tele-Health care delivering to home-dwelling persons with dementia or MCI. Based on these standards, an appropriate reimbursement scheme could be established to provide sufficiently financial incentives for long-term care providers to participate in the new healthcare delivery model in order to avoid the societal risks generated from emerging pandemics.

Third, digital inequality (defined by the disparities in the knowledge and ability of using digital and information technology across different demographic and socioeconomic groups) is a major concern due to a need for information and communication during the COVID-19 pandemic (e.g., regarding the prevention of infection and the maintenance of access to and quality of care) [46,47,48,49]. Previous studies have identified the elderly and individuals with physical health problems as groups vulnerable to digital inequality [49,50,51]. Since the Tele-Health intervention program increased the primary caregiver’s intention to use a new healthcare delivery model, it is worth noting that the Stay-at-Home-Order period has provided a teachable moment for community care providers, persons with dementia or MCI, and their primary caregivers to facilitate either a new healthcare delivery model or a new normal life in future pandemics, and in turn, to reduce the digital inequality across different demographic groups.

Fourth, though the positive effects of the Tele-Health intervention program were justified by our empirical analyses, the promotion of this new healthcare delivery model is likely to raise concerns regarding the significant substitution (negative) effects between the Tele-Health intervention program and family functioning. It is crucial to address that Taiwanese society has experienced dramatic transitions in demographics and economics [36]. The fast growth of the elderly population, together with a rapid drop in fertility and marriage rates, has resulted in a significant transformation in family structures in Taiwan. The average household size diminished from 3.58 to 2.92 persons per household over the period of 2001–2020 [52]. Although nuclear family households have remained the dominant household type (approximately 33.05% of households in 2020), the share of one-person households and two-person households without children increased from 10.73% and 13.03%, to 14.37% and 20.25%, respectively, over the same period [53]. If this tendency of family structural transition continues, we expect Taiwanese family households to gradually lose their functionality as a result of care provision for the elderly, and the same argument has been made in previous studies investigating the effect of demographic structural change on heath policy effectiveness [54,55,56]; therefore, the tradeoff between a potential loss of family functioning and a substantial reduction in the NPI intensity of persons with dementia or MCI and in their primary caregivers’ stress levels is a concern to take into account when considering the Tele-Health intervention program to cope with the societal risk of demographic transition.

### 4.2. Strengths of the Study

This study contributes to existing literature on the effects of Tele-Health intervention programs on persons with dementia or MCI in two aspects: first, this research is the first to apply the DID regression model to investigate the effects of the Tele-Health intervention program on home-dwelling persons with dementia or MCI and their primary caregivers during the COVID-19 outbreak in Taiwan. Second, the mixed effects of a single community health intervention program generated by the elderly’s participation in multiple community-based care intervention programs were eliminated by observing the effects of the Stay-at-Home-Order policy implemented during the period from May 2021 to August 2021. The results generated from this study can provide reliable evidence on the effect of the Tele-Health intervention program on home-dwelling persons with dementia or MCI and their primary caregivers in Taiwan’s long-term care system.

### 4.3. Limitations of the Study

This research, nevertheless, has several limitations. First, although valuable research data were collected during the Stay-at-Home-Order period of the COVID-19 pandemic, the non-randomization method used to collect our data results in substantial differences in some of target variables (e.g., NPI and technology acceptance attitude) across experimental and control groups. In order to accommodate unequal distributions of target variables between experimental and control groups. We evaluated whether the changes of target variables across the Stay-at-Home-Order period were different between experimental and control groups through the DID regression model. Although the goodness of fit in the DID regression model was substantiated, the small sample size (32 observations in total) prevents us from generalizing our results to the general population in Taiwan. Second, the age of the respondents would influence the results of this study. Nonetheless, the demographic characteristics of the participants and their primary caregivers were homogeneous across the experimental and control groups based on our testing results in Table 2. If the biases from the age of respondents existed, they should impact the results from experimental and control groups in the same way. The DID regression model adopted in this study could incorporate the systematic biases from the respondents. Third, we are not able to perform a concrete validation process for all instruments used in this study due to the small sample size (only 16 participants). Nevertheless, the reliability and validity of these instruments (such as the Taiwanese family functioning and technology acceptance attitude questionnaires) have been demonstrated [41,42] in previous studies. Fourth, arguments regarding whether instruments designed by researchers from Western countries are appropriate for a Taiwanese population have been made in previous studies, so the instruments used in this study were either revised versions of questionnaires from previous Taiwanese studies or established by local researchers or long-term care practitioners [41,42]; therefore, these instruments are most likely suitable for Taiwanese populations only [41,42]. The incomparability of instruments developed in previous studies [39,40,43], and those used in this study (see the Section 2), is a challenging limitation here. We recommend developing comparable or compatible instruments to measure family caregivers’ stress levels, family functioning, and attitudes regarding technology acceptance in Taiwan and other countries.

## 5. Conclusions

Society in Taiwan is subject to the risks of future pandemics and demographic transitions that potentially influence the well-being of the Taiwanese population. Considering the Stay-at-Home-Order policy implemented from May 2021 to August 2021, a period when the COVID-19 pandemic impacted the Taiwanese population tremendously, for the first time, we applied DID analyses to explore the effect of the Tele-Health intervention program on home-dwelling persons with dementia or MCI. Our results suggest that the Tele-Health intervention program significantly reduced participants’ NPI intensity and their primary caregivers’ stress levels; however, the significant substitution effects between the Tele-Health intervention program and family functioning raised concerns regarding the rapid loss of family functioning due to the extraordinary demographic transitions expected in the near future. Policymakers should consider the tradeoff between the potential loss of family functioning and the substantial improvement in the well-being of caregivers and their care recipients when considering the Tele-Health intervention program in response to the societal risks generated from pandemics and demographic transitions.

## Figures and Tables

**Table 1:** Formal parametrization of the DID regression model.

$\mathrm{Depend}\;\mathrm{Var}=Y_{it}$	Post-Intervention (*t* = 1)	Pre-Intervention (*t* = 0)	Difference
Case = 1	α + $\beta_{E}$ + $\beta_{T}$ + $\beta_{C}$ + γ$CV_{it}$	α + $\beta_{E}$ + γ$CV_{it}$	$\beta_{T}$ + $\beta_{C}$
Case = 0 (Control)	α + $\beta_{T}$ + γ$CV_{it}$	α + γ$CV_{it}$	$\beta_{T}$
Difference	$\beta_{E}$ + $\beta_{C}$	$\beta_{E}$	$\beta_{C}$

**Table 2:** Descriptive statistics.

**Panel A: Individual Charcacteristics (Variables Were Invariant across Pre- and Post-Intervention Periods)**						
**VAR**	**Description Statistics**		**All**	**Case**	**Control**	**Statistics**
			***N* = 16**	***N_p_* = 8**	***N_p_* = 8**	**[*p*-Value]**
SEX_P_	Participants’ gender.	Male	8	4	4	*χ*^2^ = 0.000
	Male = 1; Female = 0	Female	8	4	4	[1.00]
SEX_C_	Primary caregiver’s gender.	Male	4	1	3	*χ*^2^ = 1.333
	Male = 1; Female = 0	Female	12	7	5	[0.57]
MAR_C_	Primary caregiver’s marital status. Unmarried = 1; Others = 0	Unmarried	6	3	3	*χ*^2^ = 0.000
		Others	10	5	5	[1.00]
EDL_C_	Primary caregiver’s education. Bachelor or higher = 1; Others = 0	Bachelor	12	6	6	*χ*^2^ = 0.000
		Others	4	2	2	[1.00]
AGE_P_	Participants’ age.	Mean	80.375	80.625	80.125	*T* = 0.150
	Survey year (i.e., 2021) minus year of birth	(SD)	(6.46)	(7.93)	(5.14)	[0.83]
AGE_C_	Primary caregiver’s age.	Mean	57.313	56.250	58.375	*T* = −0.379
	Survey year (i.e., 2021) minus year of birth	(SD)	(10.89)	(10.36)	(11.10)	[0.71]
**Panel B: Pooling Data (Measurements Varied across the Stay-at-Home-Order Period)**						
**VAR**	**Description Statistics**		**All**	**Case**	**Control**	**Statistics**
			***N_p_* = 32**	***N_p_* = 16**	***N_p_* = 16**	**[*p*-Value]**
NPI	Intensity of neuropsychiatric symptoms measured by the NPI.	Mean	14.250	19.357	9.125	*T* = 1.981
		(SD)	(15.31)	(16.69)	(12.24)	[0.06] *
CSS	Stress scale measured by a Taiwanese family caregiver stress questionnaire.	Mean	16.531	18.750	14.313	*T* = 1.499
		(SD)	(8.54)	(9.01)	(7.68)	[0.14]
PUT	Perceived usefulness of a new health-care delivery model from the TAM survey.	Mean	16.000	17.813	14.188	*T* = 2.376
		(SD)	(4.63)	(2.46)	(5.59)	[0.02] **
PET	Perceived ease of using a new health-care delivery model from the TAM survey.	Mean	8.250	9.000	7.500	*T* = 2.038
		(SD)	(2.18)	(1.46)	(2.56)	[0.05] *
ATT	Attitude toward using a new healthcare delivery model from the TAM survey.	Mean	12.563	13.875	11.250	*T* = 2.589
		(SD)	(3.12)	(1.82)	(3.62)	[0.02] **
IUT	Intension to use a new healthcare delivery model from the TAM survey.	Mean	8.438	9.125	7.750	*T* = 1.735
		(SD)	(2.31)	(1.63)	(2.72)	[0.09] *
PS	Problem solving function from the Taiwanese family function questionnaire.	Mean	8.313	8.875	7.750	*T* = 1.275
		(SD)	(2.52)	(2.33)	(2.65)	[0.21]
CM	Communication function from the Taiwanese family function questionnaire.	Mean	5.531	5.813	5.250	*T* = 0.797
		(SD)	(1.98)	(1.80)	(2.18)	[0.43]
AF	Affective function from the the Taiwanese family function questionnaire.	Mean	15.875	16.063	15.688	*T* = 0.320
		(SD)	(3.27)	(2.67)	(3.86)	[0.75]
RL	Role function from the Taiwanese family function questionnaire.	Mean	8.406	8.375	8.438	*T* = −0.072
		(SD)	(2.41)	(2.19)	(2.68)	[0.94]
BC	Behavior control function from the Taiwanese family function questionnaire.	Mean	7.000	7.000	7.000	*T* = 0.000
		(SD)	(1.24)	(1.37)	(1.15)	[1.00]

Note: SD denotes standard deviation. An exact Fisher test was used for the homogeneity test to generate a *p*-value of *χ*^2^ statistics; *T* statistics and their corresponding *p*-values were generated based on the Levene equality test for variances. *N* and *Np* represent numbers of participants, and observations, respectively. “**” and “*” represent 5% and 10% significance levels, respectively.

**Table 3:** Descriptive statistics for the difference-in-differences of the target variables.

Variables	Groups	Post-Intervention		Pre-Intervention		Differences
		Mean	SD	Mean	SD	Mean
CSS	All	15.125	8.59	17.938	8.54	−2.813
	Case	16.375	9.87	21.125	7.99	−4.750
	Control	13.875	7.55	14.750	8.31	−0.875
	Case-Control	2.500		6.375		−3.875
NPI	All	14.063	16.42	14.438	14.66	−0.375
	Case	18.000	18.94	20.750	15.29	−2.750
	Control	10.125	13.55	8.125	11.63	2.000
	Case-Control	7.875		12.625		−4.75
ATT	All	12.688	3.53	12.438	2.76	0.250
	Case	14.250	1.49	13.500	2.14	0.750
	Control	11.125	4.36	11.375	3.02	−0.250
	Case-Control	3.125		2.125		1.000
IUT	All	8.500	2.58	8.375	2.09	0.125
	Case	9.500	1.41	8.750	1.83	0.750
	Control	7.500	3.16	8.000	2.39	−0.500
	Case-Control	2.000		0.750		1.250
PS	All	8.125	2.25	8.500	2.83	−0.375
	Case	8.125	1.64	9.625	2.77	−1.500
	Control	8.125	2.85	7.375	2.56	0.750
	Case-Control	0.000		2.250		−2.250
CM	All	5.375	1.82	5.688	2.18	−0.313
	Case	5.250	1.39	6.375	2.07	−1.125
	Control	5.500	2.27	5.000	2.20	0.500
	Case-Control	−0.250		1.375		−1.625
AF	All	15.563	3.50	16.188	3.10	−0.625
	Case	15.125	2.95	17.000	2.14	−1.875
	Control	16.000	4.14	15.375	3.81	0.625
	Case-Control	−0.875		1.625		−2.500
RL	All	8.625	2.47	8.188	2.40	0.438
	Case	8.250	2.19	8.500	2.33	−0.250
	Control	9.000	2.83	7.875	2.59	1.125
	Case-Control	−0.750		0.625		−1.375
BC	All	6.688	1.40	7.313	1.01	−0.625
	Case	6.375	1.60	7.625	0.74	−1.250
	Control	7.000	1.20	7.000	1.20	0.000
	Case-Control	−0.625		0.625		−1.250

**Table 4:** Results for the ANCOVA models.

DV = Y_t_	CSS_t_	NPI_t_	IUT_t_	ATT_t_	PS_t_	CM_t_	AF_t_	RL_t_	BC_t_
Independent Variable	Coeff (T Value)	Coeff (T Value)	Coeff (T Value)	Coeff (T Value)	Coeff (T Value)	Coeff (T Value)	Coeff (T Value)	Coeff (T Value)	Coeff (T Value)
*Case*	−3.560 (−1.53)	−5.915 (−1.43)	1.614(1.68)	1.693(1.09)	−1.376 (−1.46)	−0.987 (−1.21)	−0.955 (−0.88)	−1.265 (−1.60)	−0.289 (−0.48)
Y_t−1_	0.951 (6.75) ***	1.092 (7.51) ***	0.514 (2.13) *	0.674 (2.32) **	0.612 (3.55) ***	0.536 (2.77) **	0.940 (5.68) ***	0.825 (4.86) ***	0.646 (2.31) **
Constant	−0.146 (−0.06)	1.250 (0.44)	4.443 (2.04) *	3.458 (1.00)	3.615 (2.57) **	2.819 (2.53) **	2.476 (0.95)	2.505 (1.73)	2.954 (1.48)
SEX			−2.114 (−2.20) **						
NPI_t−1_							−0.115 (−3.04) **		−0.059 (−2.86) **
ANCOVA	*F*= 2.34 *p* = 0.15	*F* = 2.05 *p* = 0.18	*F* = 2.82 *p* = 0.12	*F* = 1.20 *p* = 0.29	*F* = 2.13 *p* = 0.17	*F* = 1.46 *p* = 0.25	*F* = 0.78 *p* = 0.39	*F* = 0.57 *p* = 0.13	*F* = 0.23 *p* = 0.64
Normality	*BJ* = 1.38 *p* = 0.50	*BJ* = 1.53 *p* = *0.46*	*BJ* = 3.20 *p* = 0.20	*BJ* = 4.83 *p* = 0.09 *	*BJ* = 1.27 *p* = 0.53	*BJ* = 0.70 *p* = 0.71	*BJ* = 0.74 *p* = 0.69	*BJ* = 2.22 *p* = 0.33	*BJ* = 2.839 *p* = 0.27
Heteroske-dasticity	*LR* = 0.84 *p* = 0.66	*LR* = 5.12 *p* = 0.08 *	*LR* = 3.30 *p* = 0.35	*LR* = 4.14 *p* = 0.13	*LR* = 4.16 *p* = 0.13	*LR* = 1.35 *p* = 0.51	*LR* = 5.754 *p* = 0.12	*LR* = 2.53 *p* = *0.28*	*LR* = 6.89 *p* = 0.08 *
*R* ^2^	78.28%	82.40%	57.20%	25.89%	49.25%	37.42%	76.42%	65.38%	55.85%
Adj-*R*^2^	75.95%	79.69%	46.50%	14.49%	41.44%	27.79%	70.53%	60.05%	44.81%

Note: “***”, “**” and “*” represent 1%, 5% and 10% significance level, respectively.

**Table 5:** Results for the difference-in-differences regression models.

Dependent Var	CSS	NPI	IUT	ATT	PS	CM	AF	RL	BC
Independent Variable	Coeff (T Value)	Coeff (T Value)	Coeff (T Value)	Coeff (T Value)	Coeff (T Value)	Coeff (T Value)	Coeff (T Value)	Coeff (T Value)	Coeff (T Value)
*Case* × *Time*	−3.875 (−1.99) *	−8.796 (−2.82) **	0.721 (2.04) *	0.241 (0.63)	−2.250 (−2.46) **	−1.625 (−1.94) *	−3.211 (−2.76) ***	−1.375 (−1.88) *	−1.476 (−2.58) **
*Case*	6.375 (1.67)	14.648 (2.22) **	−1.094 (−2.97) ***	0.263 (0.95)	2.250 (2.79) **	1.375 (1.78) *	2.795 (1.94) *	1.379 (1.38)	1.225 (2.27) **
*Time*	−0.875 (−0.68)	2.000 (0.80)	−0.277 (−1.77) *	−0.344 (−1.31)	0.750 (1.19)	0.500 (0.82)	0.464 (0.78)	1.125 (1.80) *	0.095 (0.75)
Constant	14.750 (5.37) ***	30.780 (3.25) ***	−0.915 (−2.50) **	−0.579 (−0.66)	8.613 (10.14) ***	5.769 (8.05) ***	18.083 (14.07) ***	20.830 (5.36) ***	7.774 (31.92) ***
MAR_C_					−3.300 (−4.72) ***	−2.056 (−2.75) **			−1.034 (−2.39) **
SEX_C_								2.667 (3.19) ***	
AGE								−0.174 (−3.86) ***	
CSS			0.049 (4.28) ***				−0.184 (−2.84) **		
NPI									−0.048 (−3.96) ***
PST				0.192 (2.08) *					
PET				1.002 (5.37) ***					
ATT			0.720 (23.56) ***						
AF				0.112 (2.27) **					
BC		−3.236 (−2.78) **							
Normality	*BJ* = 3.12 *p* = 0.21	*BJ* = 1.30 *p* = 0.52	*BJ* = 0.28 *p* = 0.87	*BJ* = 0.10 *p* = 0.95	*BJ* = 0.23 *p* = 0.89	*BJ* = 0.14 *p* = 0.93	*BJ* = 1.07 *p* = 0.58	*BJ* = 2.07 *p* = 0.36	*BJ* = 1.09 *p* = 0.58
Serial Corr	*DW* = 1.88	*DW* = 1.83	*DW* = 1.89	*DW* = 1.91	*DW* = 1.95	*DW* = 1.95	*DW* = 1.89	*DW* = 2.03	*DW* = 2.01
*d_u_*	1.650	1.732	1.891	1.909	1.732	1.732	1.732	1.891	1.891
(*4-d_u_*)	2.350	2.268	2.181	2.091	2.268	2.268	2.268	2.181	2.181
Heteroske-dasticity	*LR* = 20.73 *p* = 0.19	*LR* = 50.16 *p* < 0.01 ***	*LR* = 15.78 *p* = 0.47	*LR* = 25.92 *p* = 0.06 *	*LR* = 16.80 *p* = 0.40	*LR* = 30.28 *p* = 0.02 **	*LR* = 22.44 *p* = 0.53	*LR* = 33.58 *p* < 0.01 ***	*LR* = 52.54 *p* < 0.01 ***
*R* ^2^	29.91%	29.08%	94.01%	94.05%	46.72%	29.38%	29.85%	35.54%	45.47%
Adj-*R*^2^	22.40%	18.58%	92.86%	92.62%	38.83%	18.92%	19.45%	23.14%	34.98%

Note: The cross-section random effect was used to control participants’ unobserved characteristics, and White cross-section standard errors were used to compute *T* statistics. The values of *d_u_* at the 5% significance level were obtained from Savin and White’s research [45]. “***”, “**”, and “*” represent 1%, 5%, and 10% significance level, respectively.

## Data Availability

Data are available upon request to the Research Ethics Committee of Taichung Tzu Chi Hospital through Yi-Ling Lai, the first author of this study.
